# Vps34 and PLD1 take center stage in nutrient signaling: their dual roles in regulating autophagy

**DOI:** 10.1186/s12964-015-0122-x

**Published:** 2015-11-21

**Authors:** Mee-Sup Yoon

**Affiliations:** Department of Molecular Medicine, School of Medicine, Gachon University, Incheon, 406-840 Korea

**Keywords:** Autophagy, Mammalian target of rapamycin complex1 (mTORC1), Phospholipase D1 (PLD1), Vacuolar protein sorting 34 (Vps34), Amino acids

## Abstract

Autophagy is a critical pathway leading to lysosomal degradation of cellular components in response to changes in nutrient availability. Autophagy includes the biogenesis of autophagosomes and their sequential maturation through fusion with endo-lysosomes. The class III PI3 kinase Vps34 and its product phosphatidylinositol-3-phosphate (PI(3)P) play a critical role in this process, and enable the amino acid-mediated activation of mammalian target of rapamycin (mTOR), a suppressor of autophagy. Recent studies have shown that phospholipase PLD1, a downstream regulator of Vps34, is also closely involved in both mTOR activation and autophagy. This mini review summarizes recent findings in the regulation of Vps34 and PLD1 and highlights the role of these lipid-metabolizing enzymes in both mTOR activation and autophagy.

## Introduction

Eukaryotic cells have two major proteolytic systems, the ubiquitin/proteasome system and lysosomes [1]. These two processes play a part in continuous protein turnover and in the removal of proteins which no longer serve a necessary function. The proteasomal system selectively recognizes only ubiquitinated substrates, which are typically short-lived proteins. In contrast, the lysosomal system is nonselective and targets long-lived proteins and organelles that are delivered to the lysosome via autophagy [2]. Autophagy encompasses all pathways that deliver cytoplasmic materials to the lysosome [1]. Three different types of autophagy have been described in mammals to date according to the mechanisms by which cargo is delivered to lysosomes: macroautophagy, microautophagy, and chaperone-mediated autophagy [3].

Macroautophagy (hereafter referred to as autophagy) is the primary catabolic process that is activated by cellular stressors including nutrient starvation [4]. Autophagy is mediated by the autophagosome (AP), a double-membrane vesicle that expands to engulf neighboring cytoplasmic components and organelles [5]. The formation and maturation of APs is driven by the concerted action of autophagy-related proteins [6]. Mature APs fuse with lysosomes and form the autolysosome, in which luminal acid hydrolases degrade captured proteins, lipids, carbohydrates, and nucleic acids.

Autophagy is closely tied to the biogenesis and drastic remodeling of membrane-bound organelles. It is not surprising, therefore, that there is evidence linking the involvement of lipid molecules and lipid-metabolizing enzymes to autophagy. The class III PI3 kinase Vps34, one of the more well-characterized lipid enzymes in autophagy, tightly regulates the biogenesis and maturation of APs by producing phosphatidylinositol-3-phosphate (PI(3)P). Besides the fundamental role of PI(3)P in endosomal trafficking, this lipid also binds to effectors in endosomal and autophagosomal membranes, which initiates local and specific signaling. Recent studies have shown the importance of other lipid-metabolizing enzymes, such as phospholipase D (PLD). PLD1, an isoform of PLD, has been reported to function as both a negative and a positive regulator of autophagy. This review primarily focuses on recent advances in our understanding of the paradoxical role of PLD1 in regulating autophagy.

### The process of autophagy

Autophagy can be induced by the limitation of various types of nutrients such as amino acids, growth factors, energy, and oxygen [7]. Among these nutrients, the most typical trigger of autophagy is the depletion of nitrogen or amino acids in yeast and cultured mammalian cells. In the first step of AP formation, a unique membrane called the phagophore sequesters cytoplasmic constituents. Autophagy-related proteins are subsequently recruited to the phagophore, the membrane of which contains lipids from the ER, Golgi, mitochondria, and plasma membrane [8–10]. The recruitment and attachment of autophagosomal proteins to the maturing organelle leads to the formation and maturation of the AP [6, 11].

**Fig. 1:**
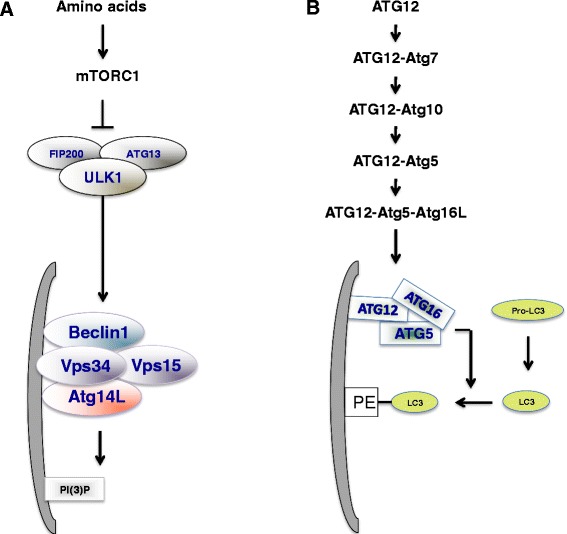
The process of autophagy. **a** Under nutrient deficient condition, ULK1 complex (ULK1-FIP200-ATG13) is activated and translocated to a particular area of the ER. Then , ULK1 activates Vps34 complex (Vps34-Vps15-Beclin1-Atg14L) to produce PI(3)P. **b** The ATG12-ATG5-ATG16L complex is crucial for formation of the covalent bond between LC3B and phosphatidylethanolamine (PE)

The first recruitment event in AP maturation and the initiation of autophagy involves activation of ULK1, a mammalian homolog of yeast Atg1 [1]. In the presence of amino acids, mammalian target of rapamycin complex 1 (mTORC1) phosphorylates Ser757 on ULK1 to inhibit kinase activity [12, 13]. When mTORC1 is blocked in amino acid-deficient conditions, ULK1 is autophosphorylated and trans-phosphorylates the binding partners ATG13 and FIP200, leading to activation of the kinase complex. The activated ULK1 complex then recruits the Vps34 complex to phagophores [14]. The Vps34 complex, which includes Vps15, Beclin1, and Atg14L, produces PI(3)P that plays a role in recruiting the phospholipid-binding proteins double FYVE-containing protein 1 (DFCP1) and WD repeat protein interacting with phosphoinoside (WIPI). DFCP1 and WIPI then promote the formation and maturation of the omegasome, an omega-shaped subdomain of the ER [15, 16]. In a separate series of events, the Atg12-Atg5-Atg16L complex is formed by the ubiquitin-like conjugation system involving Atg7 and Atg10 [17]. The direct binding of FIP200 to Atg16L is required for optimal ULK1 puncta formation, the earliest detectable events in initiation of autophagy [18, 19]. Moreover, Atg12-Atg5-Atg16L is functionally required to conjugate LC3-I to phosphoethanolamine [20], which is essential for closure of the expanding autophagosomal membrane [21]. The retention of LC3-II inside the closed autophagosome provides a critical and widely known marker of autophagy (Fig. 1).

### Rag-dependent mTORC1 activation in amino acid signaling

Mammalian target of rapamycin (mTOR) is required for repressing autophagy in amino acid-sufficient conditions. mTOR is a conserved serine and threonine kinase that exists in two biochemically and functionally distinct complexes, mTORC1 and mTORC2. mTORC1 mediates nutrient signaling, including glucose and amino acids, via phosphorylation of downstream targets such as S6K1 and 4EBP1 [22]. TSC1/2-Rheb functions as a major hub to transduce upstream signals to mTOR. TSC1/2 negatively regulates the small GTPase Rheb by acting as a GAP for Rheb which, in turn, inhibits mTORC1 activity [23].

Ras-related GTPases (Rags) have been shown, in amino acid signaling, to translocate mTORC1 to lysosomes where Rheb resides [24]. The Rag family is composed of four members (Rags A–D) that form heterodimers [24]. Rag proteins are tethered to lysosomes by Ragulator, which works as a guanine nucleotide exchange factor (GEF) for Rag A/B in amino acid-stimulated conditions [25, 26]. Amino acids activate the Rag proteins by promoting GTP-bound Rag A/B and GDP-bound Rag C/D. These activated Rag proteins bind to mTORC1 and recruit it to lysosomes, thus activating mTORC1 [24]. Notably, two parallel pathways involving mitogen- and amino acid-activation of mTORC1 converge at lysosomes in their regulation by Rheb and Rag-Ragulator, respectively [27].

### Vps34 as a dual regulator of the response to amino acid availability

Vps34 is conserved evolutionarily from yeast to mammals and was initially discovered while screening for a vacuolar protein sorting protein in yeast [28]. Vps34 also has been known to mediate amino acid sensing to activate mTORC1 signaling [29, 30]. The depletion of Vps34 blocks amino acid-stimulated mTORC1 activity, while an increase in PI(3)P levels is detected during amino acid-driven activation of mTORC1 [29, 30]. Consistent with these observations, amino acid-stimulated mTORC1 activity is dampened in Vps34 knockout mouse embryonic fibroblasts [31] and mTOR activity in Vps34 knockout embryos is drastically impaired. Vps34 regulation of mTOR likely involves other mediators. The Vps34-calmodulin complex has been suggested to enhance mTORC1 activity [32]. In addition, Vps34 activates PLD1 through the regulation of the PX domain of PLD1 [33], the implications of which will be discussed in the following section.

While Vps34 has been shown to be essential in amino acid-mediated activation of autophagy-suppressive mTOR signaling, its role as a positive regulator of autophagy has also been widely accepted. Early studies using the pharmacological PI3K inhibitors LY293002 and wortmannin implicated the involvement of Vps34 in autophagy in mammalian cells [34]. More recent investigations revealed thatVps34 constitutes several functionally distinct complexes. Vps15, a putative protein kinase, binds to Vps34 to form the defining (and bridging) component of the Vps34-Vps15 core complex, which is the major one among Vps34 complexes [35, 36]. The binding of Beclin1 to the Vps34-Vps15 complex regulates Vps34 kinase activity [37]. However, additional components are required for producing PI(3)P at correct sites and stages of autophagy. Binding of either Atg14 or UVRAG to Vps34 effectively increases the kinase activity in nutrient-deficient conditions [38]. Notably, this kinase activity is facilitated by the preferential phosphorylation of Vps34 (T163/S165) or Beclin1 (S91/S94) by AMPK and Beclin1 (S14) by ULK1 within the proautophagic Atg14/UVRAG-Vps34-Vps15 complex [38, 39].

Considering that Vps34 activity is activated in both the presence and absence of amino acids, the mechanism of Vps34 kinase activation has been somewhat perplexing until recent studies were able to distinguish the distinct regulation of individual Vps34 complexes [38, 39]. Russel et al. demonstrated that the withdrawal of amino acids activates Vps34 kinase activity in the Atg14L-containing Vps34 complex, but inhibits it in the Atg14L-free Vps34 complex [39]. In addition, Beclin1 phosphorylation by ULK1 in the Atg14L-Vps34 complex increases Vps34 kinase activity, suggesting that the presence of Atg14L determines Vps34 kinase activity in the absence of amino acids. Therefore, it is possible that specific Vps34-interacting components define the differential regulation of Vps34 complexes in amino acid signaling.

### PLD as a mediator in amino acid-induced mTOR signaling

PLD converts phosphatidylcholine to phosphatidic acid (PA), a lipid second messenger, which is required for mitogen-induced mTORC1 activation [40]. PA competes with rapamycin in binding to the FRB domain of mTOR. While several enzymes are capable of producing PA, the particular species of PA produced by PLD1 are preferentially bound by FRB domain and thereby activate mTOR kinase activity by displacing DEPTOR, an endogenous inhibitor of mTOR [41].

Our understanding of PLD1 activation by amino acids has expanded to include the upstream activation of Vps34. Amino acids activate Vps34 by a mechanism independent of the mTORC1 translocation induced by Rag-Ragulator [33]. Subsequently, the Vps34 product, PI(3)P, binds to the PX domain of PLD1, which activates PLD1 and induces its translocation to lysosomes. PLD1 translocation is dependent on Vps34 activity and the PX domain of PLD1. The depletion of Rags, P18, and raptor affects neither PLD activity nor PLD translocation, indicating that Vps34/PLD1 pathway works in parallel with Rag-Ragulator pathway [33].

mTORC1 signaling is known as a master regulator of cell growth [22]. As an upstream regulator of mTORC1, one would expect that Vps34-PLD1 axis affects cell growth. Indeed, the depletion of PLD1 and Vps34 inhibits cell growth [33], consistent with the observation that Vps34-null embryos are unviable with loss of phosphorylation of ribosomal protein S6, which is a downstream target of mTORC1 [42]. However, the requirement for Vps34-PLD1 in amino acid-induced mTOR signaling has apparently evolved in mammals, based on the observations that dVps34 mutation does not affect either cell growth or steady-state dTORC1 signaling in Drosophila [43], and the depletion of dPLD does not result in growth defects [44].

### PLD1 as a negative regulator of autophagy

One indispensable repressor of autophagy is mTORC1 [45]. In mammalian cells, mTORC1 interacts with the ULK1-Atg13-FIP200 complex and directly phosphorylates ULK1 and Atg13 [46]. mTORC1 phosphorylates ULK1 at Ser 757 to repress autophagy induction, even though the effect of ULK1 phosphorylation on the interaction between AMPK and ULK1 is opposite in two independent reports [12, 13]. Under amino acid-sufficient conditions, mTORC1 also directly phosphorylates ATG14L in ATG14L-containing Vps34 complexes to inhibit Vps34 kinase activity [47].

PLD is responsible for mTOR activation in mitogenic and amino acid signaling [33, 43, 48]. Considering its positive role in mTOR signaling, PLD has been considered a negative regulator of autophagy. Recently, Jang et al. showed that PLD1 suppresses autophagy in cancer cells [49]. PLD1 inhibition induces ULK1 Ser 555 phosphorylation by AMPK activation and suppresses ULK1 Ser 757 phosphorylation by mTOR. In addition, PLD1 overexpression decreases the interaction between Beclin1 and Vps34, an important proautophagic interaction.

Autophagy is necessary in all cells to remove damaged or long-lived proteins and organelles, and is especially so in tumor cells to relieve metabolic stress in adverse conditions. Despite the observation that autophagy suppresses tumorigenesis [50], it has been suggested that autophagy plays a positive role in tumor progression. The inhibition of autophagy by knockdown or pharmacological agents kills tumor cells [51], and autophagy was proposed to relieve tumor cells from high metabolic demand in hypoxic conditions [52]. Notably, PLD1 inhibition increased cell death in Atg7-depleted MDA-MB231 cells, suggesting that PLD inhibition sensitizes cancer cell to metabolic stress by the inhibition of autophagy [49].

### PLD as a positive regulator in autophagy

Autophagy involves substantial biogenesis and remodeling of intracellular membrane encycling intracellular organelles [53]. Diverse proposals have emerged to explain the origin of the autophagosome membranes and the site of its nucleation. These support the idea of de novo membrane synthesis as well assembly from pre-exisiting membranes such as the ER, the Golgi, endosomes, mitochondria and plasma membrane. The ER is the most feasible candidate for an initial membrane source and the platform for autophagosome formation under amino acid starvation – though the contributions from the Golgi, plasma membrane, and mitochondria are not negligible. Notably, some Atg proteins, specifically ULK1 and Atg14L, are localized to the ER membrane in the absence of nutrients [54, 55]. The scavenging of pre-existing membranes is shown to start from the omegasome of the ER, which is supported by two independent tomography studies [56, 57]. Once the double-membrane AP is formed, it can fuse with endosomes, generate amphisomes, and then, ultimately, fuse with lysosomes.

PI(3)P produced by Vps34 is involved in controlling the biogenesis and maturation of APs through PI(3)P effectors that contain either FYVE or PX domain. PLD1 has been suggested as one such PI(3)P effector, regulating autophagy through its PX domain. Dall’Armi et al. observed that PLD1 partially colocalizes with the AP marker LC3 under nutrient-starved conditions in accordance that GFP-PLD1 is enriched on the outer membrane of AP-like structures, a result of heterotypic fusion after PLD1 is translocated from endosomes to APs during the formation of APs [58]. In addition, upon nutrient deprivation PLD activity is increased, which is blocked by treatment with wortmannin [58]. Interestingly, blocking Vps34 with a pharmacological inhibitor or by genetic ablation decreases the colocalization of PLD1 with LC3, and PX-mutated PLD1 does not colocalize with LC3. These results indicate that PLD1 localization is controlled by its PX domain and Vps34. Indeed, PLD1 inhibition decreases autophagy, resulting in a small but significant decrease in the size of LC3-positive compartments and a more pronounced reduction in the total surface area of these compartments. Therefore, Dall’Armi et al. suggested that PLD1 plays a role in the AP membrane fusion and maturation steps downstream of Vps34 [58].

Arf6, a small GTPase, has been recently suggested as an upstream regulator of PLD activity in AP formation [59]. Arf6 promotes AP formation via phosphatidylinositol-4,5-bisphosphate in an Atg16L-clathrin-dependent and GRAF1-mediated manner. The Arf6 N48R mutation, which blocks PLD activity, decreased LC3-II levels in bafilomycin A1-treated cells, supporting a positive role for PLD1 in autophagy.

PLD1 has also been demonstrated to regulate autophagic flux and clearance of α-synuclein aggregates [60]. The inhibition of PLD1 resulted in the accumulation of LC3-II, p62, and ubiquitinated proteins and treatment with bafilomycin A1, an inhibitor of lysosomal acidification, did not change the level of LC3-II or p62, indicating defects in maturation of APs to autolysosomes. As a result, α-synuclein aggregates accumulated in APs in differentiated human neuroblastoma SH-SY5Y cells, leading to cell death. These results imply the significant regulation of PLD in the clearance of pathogenic protein aggregates in neurodegenerative diseases.

It has been shown that PLD and its product PA facilitate membrane trafficking, including membrane fusion [61]. PA has a conical shape that packs well in membranes with negative curvature, as would be the case with a site in the process of budding from a donor membrane [61]. PA has also been reported to promote budding from the Golgi complex, exocytosis, and endocytosis. In addition, the yeast PLD, Spo14p, is required to assemble sporulation-related prospore membranes under starvation conditions [62] and is involved in AP formation-dependent unconventional secretion of *Pichia pastoris* Acb1 [63]. Collectively, these results support the hypothesis that PLD1 is involved in membrane trafficking and the expansion of APs under nutrient-starved conditions.

## Conclusion

Autophagy provides cells with internal nutrients when external ones are deficient. When nutrients are plentiful, however, mTOR induces cell growth and inhibits autophagy, thus allowing cells to take advantage of the available resources. In order to adjust cells to cellular and external nutrient availability, both mTOR activation and autophagy have to be tightly regulated. As highlighted in this review, Vps34 and PLD1 play pleiotypic roles in the control of nutrient signaling, in terms of autophagy and mTOR activation.

While Vps34 and PLD1 regulate amino acid-induced mTOR activation, both enzymes have also been shown to be involved in the positive regulation of autophagy. Even though Vps34 and PLD1 are known to play a role in the biology of endo-lysosomes, this paradoxical regulation needs to be further clarified by studying their molecular mechanisms to differentiate their dual functions. The upstream regulation of Vps34 remains to be determined and would likely help distinguish the dual functions of Vps34 and PLD1.

The mechanism by which PLD1 regulates opposite roles in response to amino acids is still puzzling. One plausible explanation of PLD1’s dual function is the potential existence of distinct pools of PLD1 in cells. The localization of PLD1 to lysosomes is critical for mTOR activation in amino acid stimulation [33]. However, it is not clear whether PLD1 localization on other organelles plays any other biological roles. It is possible that PLD1 binds to PI(3)P on the ER or the Golgi membrane under amino acid starvation and regulates the formation of autophagosome, whereas lysosomal localized PLD1 regulates mTOR signaling in the presence of amino acids. The analysis of PLD1 subcellular localization under amino acid starvation will help address the possibility of distinct pools of PLD1 with diverse biological roles.

A recent study using Vps34-deficient mice and mouse embryonic fibroblasts showed that Vps34 does not control the basal activity of mTORC1, but is essential for acute amino acid-induced mTORC1 activation [31]. Liver-specific Vps34 knockout mice developed hepatomegaly and hepatic steatosis, which is highly similar to the phenotype observed in the autophagy-deficient Atg7−/− and Atg5−/− livers. These results confirmed the dual role of Vps34 in mTORC1 activation and autophagy. By the same token, the examination of PLD1 knockout mice would further clarify the role of PLD1 in both mTOR activation and autophagy.
